# Successful treatments with polymyxin B hemoperfusion and recombinant human thrombomodulin for fulminant *Clostridium difficile*-associated colitis with septic shock and disseminated intravascular coagulation: a case report

**DOI:** 10.1186/s40792-016-0199-5

**Published:** 2016-07-28

**Authors:** Kazuhito Minami, Yoshihisa Sakaguchi, Daisuke Yoshida, Manabu Yamamoto, Masahiko Ikebe, Masaru Morita, Yasushi Toh

**Affiliations:** 1 Department of Gastroenterological Surgery, National Kyushu Cancer Center, Notame 3-1-1, Minami-ku, Fukuoka 811-1395 Japan; 2 Department of Gastroenterological Surgery, National Hospital Organization Kyushu Medical Center, Jigyohama 1-8-1, Chuo-ku, Fukuoka 810-8563 Japan

**Keywords:** Fulminant *Clostridium difficile*-associated colitis, Septic shock, Disseminated intravascular coagulation (DIC), Polymyxin B hemoperfusion (PMX-HP), Recombinant human thrombomodulin (rhTM)

## Abstract

**Background:**

*Clostridium difficile* (CD)‐associated colitis (CDAC) is endemic and a common nosocomial enteric disease encountered by surgeons in modern hospitals due to prophylactic or therapeutic antibiotic therapies. Currently, the incidence of fulminant CDAC, which readily causes septic shock followed by multiple organ dysfunction syndromes, is increasing. Fulminant CDAC requires surgeons to perform a prompt surgery, such as subtotal colectomy, to remove the septic source. It is known that fulminant CDAC is caused by the shift from an inflammatory response at a local mucosal level to a general systemic inflammatory reaction in which CD toxin-induced mediators’ cascades disseminate. Recently, it has been proven that polymyxin B hemoperfusion (PMX-HP) improves septic shock and recombinant human thrombomodulin (rhTM) controls disseminated intravascular coagulation (DIC). In addition, clinically and basically, it has been shown that these treatments can control serous chemical mediators. Therefore, it is considered that these treatments are promising ones for patients with fulminant CDAC. In the current report, we present that these treatments without surgery contributed to the improvement of sepsis due to fulminant CDAC.

**Case presentation:**

We encountered a case who developed fulminant CDAC with septic shock and DIC after laparoscopic gastrectomy for gastric cancer. At admission to the intensive care unit, his APACHE II score was 22, which indicated an estimated risk of hospital death of 42.4 %. Our therapies were not the subtotal colectomy to remove septic sources but the combination treatments with both PMX-HP and rhTM. These combination therapies resulted in excellent outcomes, namely the dramatic improvement of septic shock and DIC and the patient’s survival. We speculate that these combination therapies completely inhibit the CD toxin-induced mediators’ cascades and correspond to the removal of septic sources.

**Conclusions:**

We recommend both PMX-HP and rhTM for patients who develop fulminant CDAC with septic shock and DIC to increase the survival benefit and replace the need for surgical treatment.

## Background

*Clostridium difficile* (CD)-associated colitis (CDAC), which is one of the common nosocomial enteric diseases encountered by surgeons, is typically due to the exposure of antibiotics and consequently endemic disease in modern hospitals [1–3]. Recently, the incidence of fulminant CDAC, which readily causes septic shock followed by multiple organ dysfunction syndromes (MODS), is increasing [4–7]. Fulminant CDAC often requires surgeons to perform a prompt invasive surgical treatment, such as a subtotal colectomy, in order to remove the septic source and improve the patient’s fatal situation [7–19].

Recently, it has been proven that polymyxin B hemoperfusion (PMX-HP) improves septic shock [20–23] and recombinant human thrombomodulin (rhTM) controls disseminated intravascular coagulation (DIC) [24–29]. In addition, clinically and basically it has been shown that these treatments can control serous chemical mediators. On the other hand, it is known that fulminant CDAC with MODS is caused by the shift from an inflammatory response at a local mucosal level to a general systemic inflammatory reaction in which CD toxin-induced mediators’ cascades disseminate [30–36]. Therefore, it is considered that these treatments are promising ones for patients with fulminant CDAC.

In the current report, we present that these treatments without surgery contributed to the improvement of sepsis due to fulminant CDAC.

## Case presentation

**Fig. 1:**
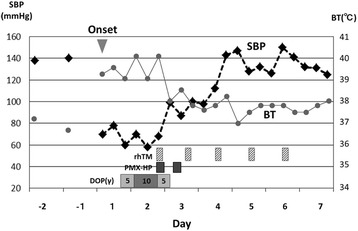
Clinical course of the vital signs and treatments. Abbreviations: *SBP* systolic blood pressure, *BT* body temperature, *DOP* dopamine, *PMX-HP* polymyxin B hemoperfusion, *rhTM* recombinant human thrombomodulin

**Fig. 2:**
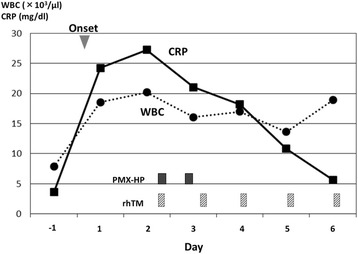
Clinical course of the WBC count, serum CRP level, and treatments. Abbreviations: *WBC* white blood cell, *CRP* C-reactive protein, *PMX-HP* polymyxin B hemoperfusion, *rhTM* recombinant human thrombomodulin

**Fig. 3:**
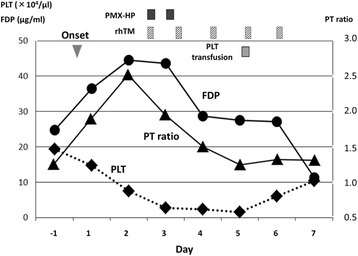
Clinical course of the PLT count, blood FDP level, blood PT level, and treatments. Abbreviations: *PLT* platelet, *FDP* fibrin degradation product, *PT* prothrombin time, *PMX-HP* polymyxin B hemoperfusion, *rhTM* recombinant human thrombomodulin

**Fig. 4:**
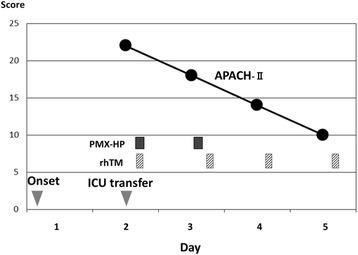
Clinical course of the APACHE II score and treatments. Abbreviations: *PMX-HP* polymyxin B hemoperfusion, *rhTM* recombinant human thrombomodulin, *APACHE II* Acute Physiology and Chronic Health Evaluation II

A 51-year-old male patient who underwent laparoscopic partial gastrectomy for early gastric cancer had been given intravenous cefazolin for 2 days preventively and 5 days after the surgery suddenly developed a high-grade fever (over 39 °C) and severe diarrhea. We immediately administered oral vancomycin (VCM), Lac-B, viz. probiotics; and enough extracellular fluid because we empirically suspected that these symptoms were due to CDAC or methicillin-resistant *Staphylococcus aureus*-associated enteritis. A diagnosis of CDAC was rapidly made by confirming the presence of toxin A in his feces. Although these medications were initiated, 24 h after the onset the patient developed septic shock requiring vasopressor agents and MODS composed of DIC and acute renal failure (ARF). When he was transferred to the intensive care unit (ICU), his Acute Physiology and Chronic Health Evaluation (APACHE) II score [37] was 22, which estimated his risk of hospital death to be 42.4 % (Table 1). According to the clinical and radiological findings, he did not have any colonic perforation or toxic megacolon; thus, we avoided an invasive surgery (such as subtotal colectomy) but alternatively treated him using both PMX-HP to improve septic shock [20–23] and rhTM to control DIC [24–29]. In the first 6 h after starting both treatments, his systolic blood pressure (SBP) improved, requirement for the vasopressor agent decreased, and body temperature (BT) dropped by approximately two degrees. Twenty-four hours after the treatments, septic shock was dramatically improved (Fig. 1). Three days after the treatments, an improvement in severe inflammation was noted according to white blood cell (WBC) count and C-reactive protein (CRP) level (Fig. 2) and an improvement in DIC according to the fibrin degradation product (FDP) level and prothrombin time (PT) (Fig. 3). A temporary decline in the platelet count was controllable with platelet transfusion (Fig. 3). Although four cycles of continuous hemodiafiltration (CHDF) were necessary as a replacement therapy to ARF, the further progression of MODS was not observed and the APACHE II score satisfactorily decreased daily (Fig. 4). Although clinically moderate diarrhea and a mild fever were observed, his general condition also improved. Consecutive toxin A tests, except the first one, were all negative. Five days after the treatments, the patient overcame fulminant CDAC through the use of the abovementioned therapies. Throughout the entire clinical course, neither endotoxemia nor bacteremia was observed.

**Table 1:** Vital signs, APACHE II score, and laboratory data at the time of ICU transfer

Body temperature (°C)	40	Ht (%)	43.8
Symbolic blood pressure (mmHg)	80	WBC (/μl)	18500
Median blood pressure (mmHg)	43	PLT (×10^4^/μl)	14.8
Administration of dopamin (*γ*)	10	T. bil (mg/dl)	3.4
Heart rate (/min)	150	Na (mEq/l)	137
Respiratory rate (/min)	32	K (mEq/l)	4.4
Urine output (ml/h)	5	Scr (mg/dl)	5.21
Glasgow coma scale	15	CRP (mg/dl)	24.2
APACHE II score	22	PT (%)	55
Estimated risk of hospital death (%)	42.4	FDP (μg/ml)	36.5
		pH	7.38
		PaO_2_/FiO_2_ (mmHg)	322

Abbreviations: *APACHE II* Acute Physiology and Chronic Health Evaluation II, *Ht* hematocrit, *WBC* white blood cell, *PLT* platelet, *T. bil* total bilirubin, *Na* serum sodium, *K* serum potassium, *Scr* serum creatinine, *CRP* C-reactive protein, *PT* prothrombin time, *FDP* fibrin degradation products, *pH* hydrogen ion concentration, *PaO*_*2*_ airway opening pressure, *FiO*_*2*_ fraction of inspired oxygen

### Discussion

Currently, CDAC is endemic and a common nosocomial enteric disease encountered by surgeons in modern hospitals due to prophylactic or therapeutic antibiotic therapies [1–3]. Recently, both the incidence and the severity of CDAC have been increasing, and one possible explanation for these increases is the emergence of highly toxigenic and lethal strains of CD [4–7]. The above shows the need for surgeons to consider more serious treatment against CDAC. In fulminant CDAC, which has a higher lethal rate, it is especially necessary for surgeons to promptly decide whether or not to perform an invasive surgical treatment, such as subtotal colectomy, which means the removal of the septic sources and probable improvement of the patients’ ill condition [7–19].

In our case that suddenly developed fulminant CDAC with septic shock requiring vasopressor agents and MODS composed of DIC and ARF, prompt surgical treatment in order to remove the septic sources was recommended. However, we alternatively treated the patient with both PMX-HP and rhTM therapies. The reason for having chosen these treatments is as follows: (1) there was neither colonic perforation nor toxic megacolon, which absolutely requires surgery; (2) PMX-HP is an effective extracorporeal blood purification treatment for improving septic shock [22]; and (3) rhTM can effectively inhibit systemic dissemination of intravascular coagulation [24–29]. The combination therapies produced excellent outcomes in this case, namely the dramatic improvement of septic shock and DIC, the inhibition of MODS progression, and the patient’s survival. We speculate that the two below-mentioned factors corresponded to the removal of the septic source, namely as result of the surgical treatment. First, oral VCM medication could suppress CD’s proliferation and the further production of CD toxins. Second, both PMX-HP and rhTM could completely inhibit the CD toxin-induced mediators’ cascades. This notion is based on the following evidence. First, fulminant CDAC with MODS is caused by the shift from an inflammatory response at a local mucosal level to a general systemic inflammatory reaction in which CD toxin-induced mediators’ cascades disseminate [30–36]. Second, although PMX-HP removes circulating endotoxin by adsorption and theoretically prevents the progression of the biological cascade of sepsis, several studies and published reports have demonstrated that PMX-HP can reduce the plasma levels of cytokines and sepsis-related factors, namely TNF-α, IL-6, IL-10, *N*-arachidonoylethanolamine (AEA), 2-arachidonoyl glycerol (2-AG), and high-mobility group box-1 (HMGB-1) [21, 23, 38, 39]. Indeed, there were case reports published which showed that PMX-HP decreases the serum levels of endogenous cannabinoids (anandamide and 2-AG) and inflammatory cytokine (IL-6) in parallel with the clinical improvement of fulminant CDAC [40, 41]. Third, many studies and fundamental researches have shown that rhTM also has an anti-inflammatory ability through both the activated protein C and the lectin-like domain-dependent pathway [42–46]. In particular, the thrombin-rhTM complex demonstrates an anti-inflammatory ability through neutralizing HMGB-1 [47, 48], which is known to be a mediator of lethality and is released from necrotic cells or macrophages/activated dendritic cells with potent pro-inflammatory function, which in turn causes shock or MODS when being disseminated in the systemic circulation [49–51]. Finally, septic shock and MODS in our case were not induced by endotoxemia or bacteremia, and a dramatic improvement was observed immediately after the initiation of the combination therapies.

## Conclusions

Both PMX-HP and rhTM therapies for patients who develop fulminant CDAC with septic shock and DIC can provide survival benefits and replace the need for invasive surgical treatments to remove the septic sources.

## Consent

Written informed consent was obtained from the patient for publication of this case report and any accompanying images. A copy of the written consent is available for review by the Editor-in-Chief of this journal.

## Abbreviations

APACHE II, Acute Physiology and Chronic Health Evaluation II; BT, body temperature; CD, *Clostridium difficile*; CDAC, *Clostridium difficile*-associated colitis; CHDF, continuous hemodiafiltration; CRP, C-reactive protein; DIC, disseminated intravascular coagulation; FDP, fibrin degradation product; MODS, multiple organ dysfunction syndromes; PMX-HP, polymyxin B hemoperfusion; rhTM, recombinant human thrombomodulin; SBP, systolic blood pressure; VCM, vancomycin; WBC, white blood cell
